# Supplementary material for: Diguanylate Cyclase GdpX6 with c-di-GMP Binding Activity Involved in the Regulation of Virulence Expression in Xanthomonas oryzae pv. oryzae
Source: Microorganisms. 2021 Feb 26;9(3):495. doi: 10.3390/microorganisms9030495 (PMC7996900; doi:10.3390/microorganisms9030495)
Supplement: Supplementary file 1 [file microorganisms-09-00495-s001.pdf]

Diguanylate cyclase GdpX6 with c-di-GMP binding activity involved in the regulation of the expression of virulence in *Xanthomonas oryzae* pv. *oryzae*

Weiwei Yan[1,2], Yiming Wei[1], Susu Fan[3], Chao Yu[1], Fang Tian[1], Qi Wang[2], Fenghuan Yang[1*], Huamin Chen[1]

[1] State Key Laboratory for Biology of Plant Diseases and Insect Pests, Institute of Plant Protection, Chinese Academy of Agricultural Sciences, Beijing 100193, China; yww_vickey@163.com (W.Y.); weiym132@163.com (Y.W.); yuchao@caas.cn (C.Y.); tianfang@caas.cn (F.T.); chenhuamin@caas.cn (H.C.)

[2] The MOA Key Laboratory of Plant Pathology, Department of Plant Pathology, College of Agronomy and Biotechnology, China Agricultural University, Beijing, China; wangqi@cau.edu.cn

[3] Shandong Provincial Key Laboratory of Applied Microbiology, Ecology Institute, Shandong Academy of Sciences, Jinan 250014, Shandong Province, China; 1986fansusu@163.com

* Correspondence: yangfenghuan@caas.cn (F.Y.); Tel.: +86-010-62896063

Table S1 Primers used in this study

Primer	Sequences (5′-3′)	Restriction enzyme/
gdpX6LF	CG**GGATCC**GCCCGATCCGCCGACCTCG	*Bam*H/gene deletion
gdpX6LR	CG**GAATTC**TGTGGCTACCTGTCTGGA	*EcoR* I / gene deletion
gdpX6RF	CG**GAATTC**GCCTTCAGGCGGTTCGGT	*EcoR* I /gene deletion
gdpX6RR	AA**CTGCAG**AAGTCATCCTCGTCGGCA	*Pst* I / gene deletion
gdpX6CF	GG**GGTACC**TCCAGACAGGTAGCCACAGTGAC	*Kpn* I / complemention
gdpX6CR	CG**GGATCC**AGCCGACGTCCGATTCC	*Bam*H I /complemention
GdpX6PF	CTC**GGTACC**CTCGAGGGATCCATGGACCTGCAACTGCGCC	*Bam*H I / protein expression
GdpX6PR	AC**AAGCTT**CTAAGCCGACGTCCGATTCC	*Hind*III /protein expression
$E^{411}F$	GCAGAGTTCGGCATGATCCTGCC	point mutation
$E^{411}R$	AGGATCATGCCGAACTCTGCGCCGCCGTAGCGCGC	point mutation
$D^{403}F$	TCGCGACGCAGCGAAGCCGTGGCCGCG	point mutation
$D^{403}R$	ACGGCTTCGCTGCGTCGCGAGCAGGCCGC	point mutation

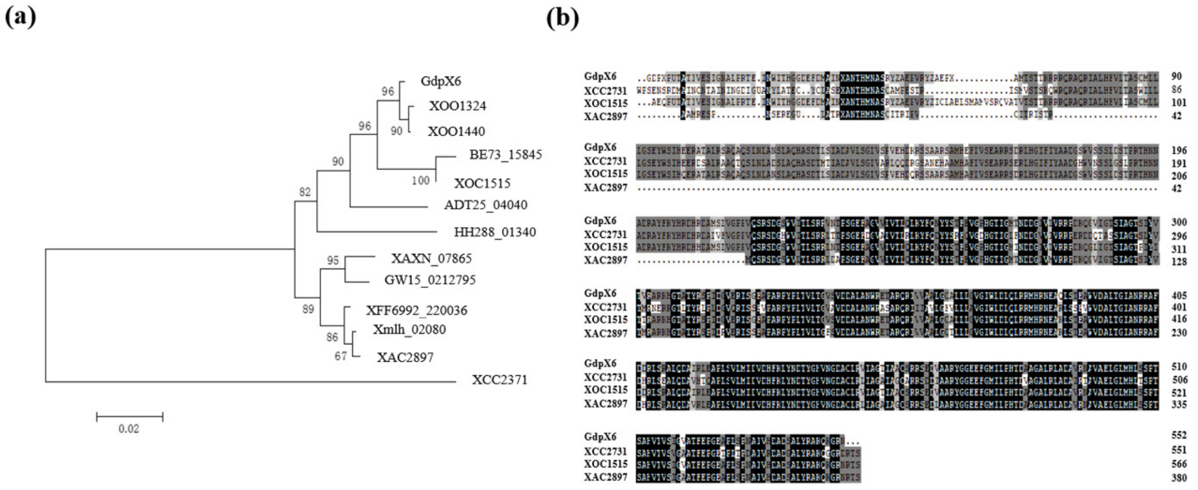

Figure S1: Sequence analysis of the homologs of GdpX6 from *Xanthomonas* species. (a) The neighbor-joining tree reconstructed based on the sequences of homologs of GdpX6 from *Xanthomonas* species by MEGA (version 7.0.21). (b) Sequence alignment of GdpX6 with the proteins XCC2731, XOC1515 and XAC2897 by using the software DNAMAN. Black and gray highlighting show amino acid residues with100% and ≥75% homology, respectively

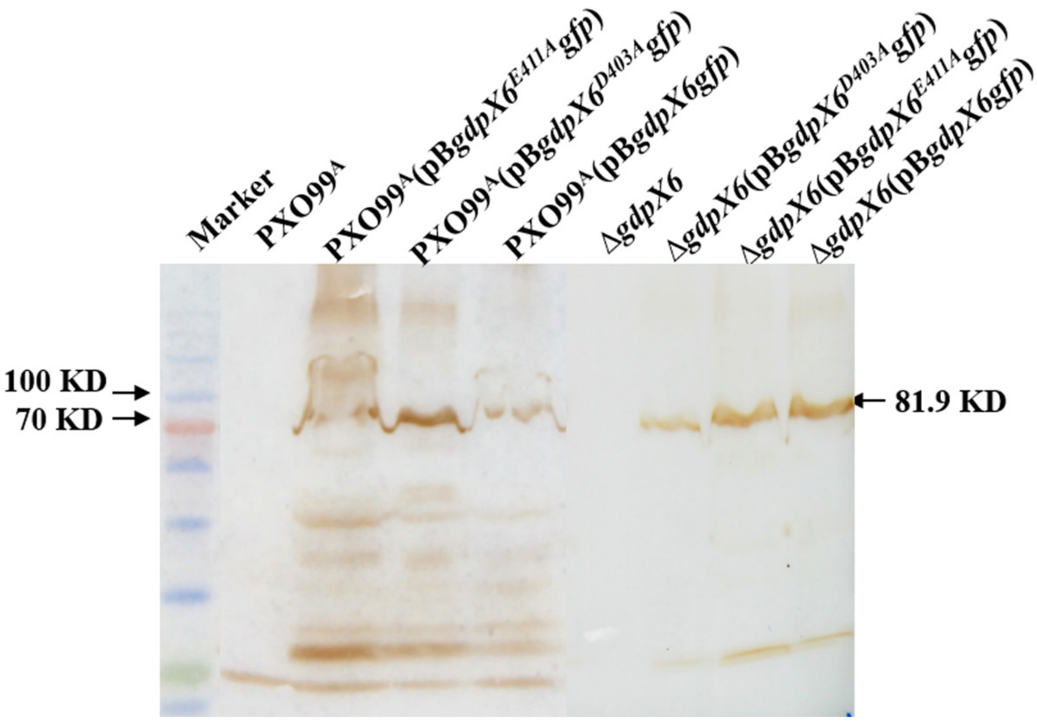

Figure S2: Western blot analyses showing expression of GFP fusion proteins with GdpX6, GdpX6$^{GGDEF-D403A}$, GdpX6$^{GGEDF-E411A}$ in *Xoo* strains. A total of 10 µg proteins were loaded in each lane and analyzed by immunoblotting with anti-GFP antibodies. All individual blots were repeated three times using samples from independent cultures.

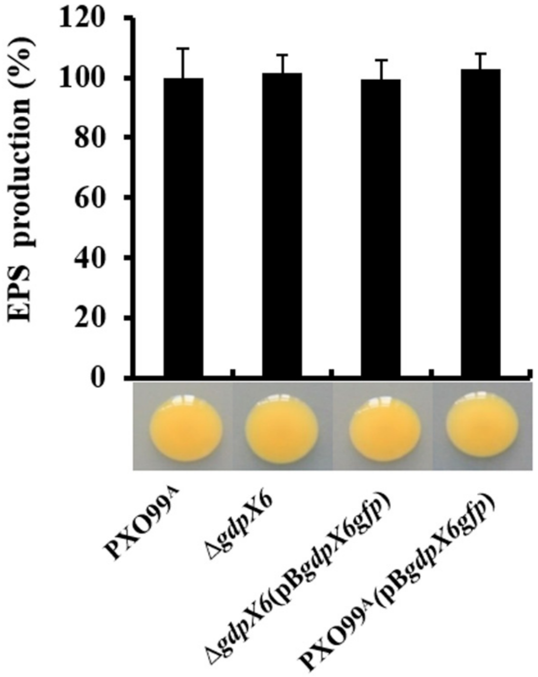

Figure S3. GdpX6 does not affect EPS production of *Xoo*. The EPS production of all *Xoo* strains were determined by ethanol precipitation method. No significant difference was observed between PXO99^{A} and Δ*gdpX6*, ΔgdpX6(pB*gdpX6gfp*), PXO99^{A} (pB*gdpX6gfp*) in terms of EPS production by quantification. All experiments were performed thrice in triplicate. The error bar represents standard deviations.

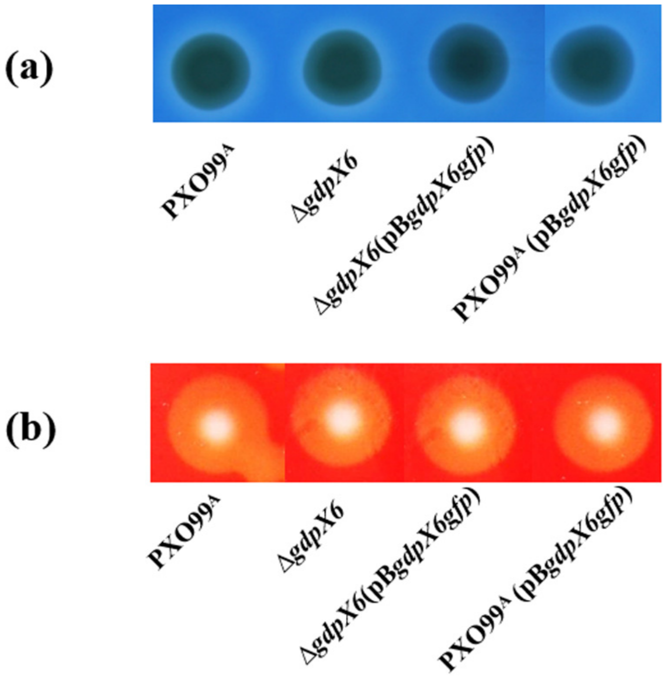

Figure S4: GdpX6 does not regulate xylanase and cellulase activities of *Xoo*. The xylanase and cellulase activities were assayed on the PSA plates with 0.2% RBB-xylan and 0.5% carboxymethyl cellulose, respectively. No significant difference in cellulase and xylanase activities of PXO99^{A} and Δ*gdpX6*, Δ*gdpX6*(pB*gdpX6gfp*), PXO99^{A}(pB*gdpX6gfp*) was found. All experiments were performed thrice in triplicate.
